# The safe motherhood referral system to reduce cesarean sections and perinatal mortality - a cross-sectional study [1995-2006]

**DOI:** 10.1186/1742-4755-8-34

**Published:** 2011-11-23

**Authors:** Marilza VC Rudge, Izildinha Maestá, Paula MSS Moura, Cibele VC Rudge, Glilciane Morceli, Roberto AA Costa, Joelcio Abbade, José C Peraçoli, Steven S Witkin, Iracema MP Calderon, Collaborative group

**Affiliations:** 1 Department of Gynecology and Obstetrics, Botucatu Medical School, Sao Paulo State University/UNESP, Brazil

**Keywords:** Referral system, antenatal/intrapartum care, cesarean section, perinatal mortality

## Abstract

**Background:**

In 2000, the eight Millennium Development Goals (MDGs) set targets for reducing child mortality and improving maternal health by 2015.

**Objective:**

To evaluate the results of a new education and referral system for antenatal/intrapartum care as a strategy to reduce the rates of Cesarean sections (C-sections) and maternal/perinatal mortality.

**Methods:**

*Design*: Cross-sectional study. *Setting*: Department of Gynecology and Obstetrics, Botucatu Medical School, Sao Paulo State University/UNESP, Brazil. *Population*: 27,387 delivering women and 27,827 offspring. *Data collection*: maternal and perinatal data between 1995 and 2006 at the major level III and level II hospitals in Botucatu, Brazil following initiation of a safe motherhood education and referral system. *Main outcome measures*: Yearly rates of C-sections, maternal (/100,000 LB) and perinatal (/1000 births) mortality rates at both hospitals. *Data analysis*: Simple linear regression models were adjusted to estimate the referral system's annual effects on the total number of deliveries, C-section and perinatal mortality ratios in the two hospitals. The linear regression were assessed by residual analysis (Shapiro-Wilk test) and the influence of possible conflicting observations was evaluated by a diagnostic test (Leverage), with *p *< 0.05.

**Results:**

Over the time period evaluated, the overall C-section rate was 37.3%, there were 30 maternal deaths (maternal mortality ratio = 109.5/100,000 LB) and 660 perinatal deaths (perinatal mortality rate = 23.7/1000 births). The C-section rate decreased from 46.5% to 23.4% at the level II hospital while remaining unchanged at the level III hospital. The perinatal mortality rate decreased from 9.71 to 1.66/1000 births and from 60.8 to 39.6/1000 births at the level II and level III hospital, respectively. Maternal mortality ratios were 16.3/100,000 LB and 185.1/100,000 LB at the level II and level III hospitals. There was a shift from direct to indirect causes of maternal mortality.

**Conclusions:**

This safe motherhood referral system was a good strategy in reducing perinatal mortality and direct causes of maternal mortality and decreasing the overall rate of C-sections.

## Background

In 2000, the eight Millennium Development Goals (MDGs) [1] set targets for reducing child mortality and improving maternal health by 2015. There is growing consensus that a primary bottleneck in achieving MDG is a health system that is too fragile and fragmented to deliver the appropriate volume and quality of services to those in need [2]. Development of more responsive health systems appears to be a prerequisite to achieving health-related MDGs.

The implementation of specific new interventions would enable health systems to respond better to the MDGs and make them more attainable. The provision of high-quality pregnancy and delivery care, including emergency obstetric assistance, is central to decreasing the maternal and neonatal mortality ratios. Not only does the maternal mortality rationeed to be decreased, but also direct etiologies of maternal mortality need to be identified and addressed in order to evaluate the quality of obstetric care [3].

Maternal and newborn health care currently constrains the health system. Improving access to emergency obstetric care is an effective strategy to improve pregnancy safety. To increase access to and utilization of services, interventions that implement maternal and newborn health assistance programs are needed. Within this framework, a two-level referral system was established in a low-income region of Brazil with a history ofvery high hospital maternal mortality ratio (HMMR) of 422/100,000 live births [4].

The Referral of Maternal and Perinatal Care, a safe motherhood operational study carried out in Botucatu, Brazil, aimed to develop a health program to improve maternal and perinatal outcomes. The major goals of this program were to create a system where obstetric need was based on the severity of obstetric complications, to collect and process maternal and neonatal data to establish a new database, to train medical personnel to use this program as a model and to standardize obstetric and neonatology procedures to unify diagnoses management and clinical programs for physicians and nurses. The expected results of this program were decreases in maternal and perinatal mortality ratesand in C-section rates.

The objective of this study is to evaluate the results of this safe motherhood referral system for antenatal/intrapartum care.

## Methods

This operational research was approved by the Ethics Committee of Botucatu Medical School, Sao Paulo State University-UNESP [project number 2048/2006].

### Design and population study

For this cross-sectional study, all delivery outcomes between 1995 and 2006 were analyzed for two referral hospitals located in Botucatu/SP, southeastern Brazil: Botucatu University Hospital (Level III) and Sorocabana Association Hospital (Level II), which together serve a population of about 1,200,000 people. All pregnant women were followed from delivery until puerperium and all infants from birth to discharge.

### Safe motherhood referral system

In 1995 a safe motherhood referral system for antenatal/intrapartum care was created to improve maternal and perinatal outcomes and to avoid overcrowding in the Level III hospital, which is the only referral center for high-risk pregnant women in the area. Thus, when risk factors were identified during labor or in the postpartum period, a woman is referred to the Level III hospital. Emergency obstetric care at the Level III hospital was always available to all high-risk pregnant women.

The strategy for this initiative was to create a health system structure based on the exchange of patients from the Level II to the Level III hospital appropriate to the level of care required, including the provision of effective transport from one hospital to the other. This strategy consisted of service availability, referral and other communication systems, transport between the Level II to the Level III hospital and additional financial support for hospital staff. As a result, all high-risk pregnant women were planned to deliver at the Level III hospital, and all low-risk pregnant women were planned to deliver at the Level II hospital.

A separate cadre of better-paid health workers with more specified responsibilities was established. The health worker cadre was comprised of obstetricians, anesthesiologists, neonatologists, residents and medical and nursing students. Supplemental compensation for this health care was provided by the Health Secretary of São Paulo State, Brazil.

### Data collection

Maternal and perinatal outcomes in both hospitals were analyzed according to referral status. Data were obtained using linked hospital delivery and birth logs, patient medical records and necropsy reports or death certificates. The following variables were assessed yearly: number of deliveries, delivery route, perinatal mortality rate and maternal mortality ratio. Direct and indirect causes of maternal mortality were also determined.

### Definitions

The regional maternal and perinatal services are essentially an institution-based hierarchical system with the Level III hospital at the top. The two hospitals involved in the present study are identified by level of care based on the complexity of the treatments they provide. The level III (higher complexity) hospital provides complete maternity and neonatal care, intrapartum and neonatal intensive care, transport services, outreach education services, maternal and perinatal collection and analysis and evaluation of new technologies. The level II (lower complexity) hospital provides 24-hour in-house anesthesia services, 24-hour clinical laboratory and radiology services and stabilization and transfer of complicated obstetric cases, including threatened preterm deliveries up to34 weeks [5].

HMMR was defined as the number of maternal deaths per year divided by the number of live births per year, expressed per 100,000 live births (LB). The definition of direct maternal death included death of the mother resulting from obstetrical complications of pregnancy, labor, or puerperium and from interventions, omissions, incorrect treatment, or a chain of events resulting from any of these factors. Indirect maternal death was defined as a maternal death not directly due to an obstetric cause but resulting from a previously existing disease or a disease that developed during pregnancy, labor, or puerperium that was aggravated by the maternal physiological adaptation to pregnancy.

Perinatal mortality rate (PMR) was defined as the number of deaths in the first week of life (early neonatal deaths) plus fetal deaths (stillbirths) divided by the total number of births, expressed per 1000 births [6].

### Data analysis

Simple linear regression models were adjusted to estimate the referral system's annual effects on the total number of deliveries, C-section rates, and perinatal mortality rate in the two hospitals. The theoretical assumptions for the application of simple linear regression were assessed by residual analysis (Shapiro-Wilk test). In addition, the influence of possible conflicting observations on estimate accuracy was evaluated by a diagnostic test (Leverage). Effects were considered statistically significant when *p *< 0.05.

## Results

Between 1995 and 2006, in both hospitals, there were 27,387 deliveries with 27,827 offspring (418 twin pregnancies and 11 triple pregnancies), 30 maternal deaths and 660 perinatal deaths. Among these deliveries, 10,206 deliveries were C-sections.

**Figure 1:**
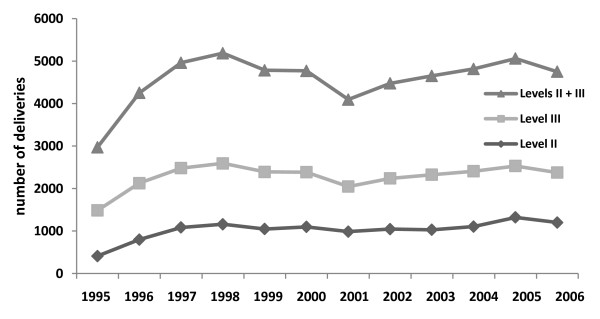
**Number of total deliveries per year at the Level II and Level III hospitals**.

**Figure 2:**
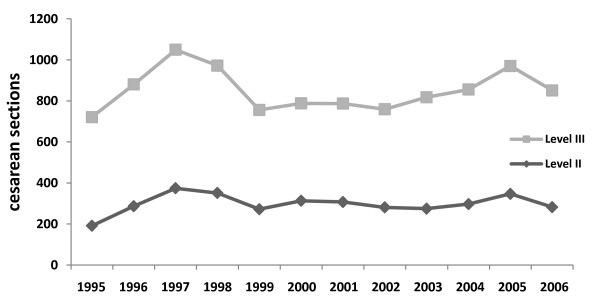
**Cesarean section deliveries per year at the Level II and Level III hospitals**.

The overall C-section rate was 37.3%: 29.1% at the Level II hospital and 43.9% at the Level III hospital. The overall PMR was 23.7 per 1000 total births, corresponding to 5.4 per 1000 total births at the Level II hospital and 38.3 per 1000 total births at the Level III hospital. The delivery of twin fetuses occurred more frequently at the Level III hospital (2.6% vs. 0.2%), and all triplet deliveries took place at the Level III hospital. The overall HMMR was 109.5 per 100,000 live births. The HMMR [7] was low at the Level II hospital (16.3 per 100,000 live births) and very high at the Level III hospital (185.1 per 100,000 live births) (Figure 1). Thus, the C-section rate, PMR and HMMR were much higher at the Level III hospital than at the Level II hospital (Table 1; Figure 2). There was a statistically significant average annual increase by 44 births in Level II hospital (*p *= 0.011) different from the Level III hospital, where the number of births did not change significantly (*p *= 0.501). A diagnostic analysis found no value that could significantly influence the accuracy of the estimates.

**Table 1:** Total number of deliveries, Cesarean sections, hospital maternal mortality ratio (HMMR) and perinatal mortality rate (PMR) at the Level II and Level III hospitals between 1995 and 2006

	Level II	Level III	Total	*p*
Deliveries^a^	12283 (44.8)	15104 (55.2)	27387 (100)	**<0.0001**
1995 - 1998	3451	5235	8686	
1999 - 2002	4176	4886	9062	
2003 - 2006	4656	4983	9639	
C-section^a^	3577 (29.1)	6629 (43.9)	10206 (37.3)	**<0.001**
1995 - 1998	1203	2419	3622	
1999 - 2002	1173	1917	3090	
2003 - 2006	1201	2293	3494	
H-MMR^b^	2/12262 (16.3)	28/15158 (184.7)	30/27420 (109.4)	**<0.001**
1995 - 1998	0/3443	3/5207	3/8650	
1999 - 2002	2/4168	10/4896	12/9064	
2003 - 2006	0/4651	15/5055	15/9706	
PMR^c^	66/12311 (5.4)	594/15516 (38.3)	660/27827 (23.7)	**<0.001**
1995 - 1998	27/3462	255/5357	282/8819	
1999 - 2002	26/4187	166/4996	192/9183	
2003 - 2006	13/4662	173/5163	186/9825	

^a ^Number and percentage (%)

^b ^HMMR = Hospital Maternal Mortality Ratio [maternal deaths/total live birth; expressed in/100,000 LB]

^c ^PNMR = Perinatal Mortality Rate [perinatal deaths/total birth; expressed in/1000 births]

The C-section rate at the Level II hospital decreased significantly over the study period, from 46.5% in 1995 to 23.4% in 2006 (*p *= 0.001) (Figure 2). An analysis conducted on data collected during this time period using simple linear regression showed a significant effect of time on the reduction in C-section rates. Every year, there was an average decrease of 1.4% (from 0.7% to 2%, 95% CI). The normality of the residual values was checked with a Shapiro-Wilk test, which yielded a *p*-value of 0.494.

The C-section rate at the Level III hospital remained about the same over the study period: 49.2% in 1995 and 48.4% in 2006 (*p *= 0.884) (Figure 2). An analysis conducted on data collected between 1995 and 2006 using simple linear regression showed no significant effect of time on the percentage of C-sections. The normality of the residual values was checked with the Shapiro-Wilk test, which yielded a *p*-value of 0.801.

**Figure 3:**
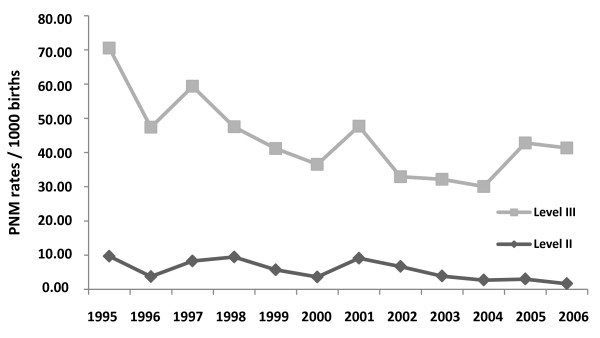
**Perinatal mortality rate per year at the Level II and Level III hospitals**.

PMR at the Level II hospital decreased significantly, from 9.71 per 1000 births in 1995 to 1.66 per 1000 births in 2006 (*p *= 0.018) (Figure 3). An analysis using simple linear regression showed a significant effect of time on the reduction of the perinatal mortality rate. Every year, there was an average decrease of 0.665 (from 0.112 to 0.963, 95% CI for the annual effect). The normality of the residual values was checked with the Shapiro-Wilk test, which yielded a *p*-value of 0.993.

PMR at the Level III hospital decreased significantly, from 60.8 deaths per 1000 births in 1995 to 39.6 per 1000 births in 2006 (*p *= 0.027) (Figure 3). A simple linear regression showed a significant effect of time on percentage reduction. Every year, there was an average decrease of 0.634 (from 0.24 to 3.27, 95% CI for the annual effect). The normality of the residual values was checked with the Shapiro-Wilk test, which yielded a *p*-value of 0.067. Required presuppositions as to the validity regression models were tested and no violation was identified. All requirements for validity of the model were met.

During the study period, hospital maternal mortality ratios (HMMR) were 16.3/100,000 in the Level II hospital and 185.1/100,000 in the Level III hospital, with little or no yearly variation in either hospital. In the Level III hospital, the average maternal death rate was 2.33 ± 1.96/year while in the Level II hospital, only two deaths occurred between 1995 and 2006. Data on 30 maternal deaths were included in the present study, 28 of which occurred at the Level III hospital. Maternal deaths due to indirect obstetric causes occurred in 13 cases: cardiopathy (4), cancer (4), anesthesia accident (1), chronic hypertension (1), hyperthyroidism (1), idiopathic lung interstitial disease (1) and cholangitis due to biliary lithiasis (1). Maternal deaths due to direct obstetric causes occurred in 17 cases: preeclampsia (7), puerperal infection (4), obstetric hemorrhage (4) and HELLP syndrome (2). The percent of maternal deaths due to indirect obstetric causes was 43.3%, and the percentage due to direct obstetric causes was 56.7% (95% CI: 38.9% to 74.4%). No significant difference was found between indirect and direct obstetric maternal death.

## Discussion

The implementation of a safe motherhood referral system for maternal and perinatal health care was shown to be associated with a decrease in C-section rate and PMR at the Level II hospital. It also decreased PMR at the Level III hospital and stabilized the C-section rate. The equalization of direct and indirect obstetric causes of maternal mortality was also a significant improvement.

The excessive use of C-sections is a serious problem in Brazil, where rates are usually above 40% [8]. C-sections are known to be associated with a higher rate of maternal complications [9,10]. They result in rehospitalization for wound complications and infection, and the average initial hospital cost for a C-section delivery is higher than for the average vaginal birth [10]. The decrease in the C-section rate observed at the Level II hospital demonstrates that it is possible to reverse the rising C-section rates in developing countries and, thereby, reduce maternal morbidity [11].

Perinatal mortality is a sensitive indicator of the quality of obstetric and neonatal care [12]. According to the World Health Organization (WHO) [6], 98% of perinatal deaths occur in developing countries. In Brazil, the few available studies on perinatal death report a rate two- to three-fold higher than that observed in developed countries [12-14]. Indirect obstetric causes of death are considered not preventable and are more frequent in developed countries [15]. In Brazil, direct obstetric causes account for most maternal deaths; preeclampsia predominates, followed by obstetric hemorrhage and puerperal infection [16]. The referral system assessed herein did not cause a reduction in the maternal mortality ratio. Nonetheless, the equalization of the direct and indirect obstetric causes of maternal mortality showed that obstetric care improved over the course of the study. To allow comparison of data during the period this fact was not described in Brazil [16].

This program is an effective two-level, parallel system whose focus was to improve maternal and perinatal outcomes, strengthen the healthcare system and removes the barriers between obstetric and perinatal care at Level II and Level III hospitals. This strategy could be considered for use in other regions as an intervention for improving the safety of pregnancy [17].

Although effective as an example to aid planning by individual governments and financial supporting agencies there are some possible limitations of this type of study. The major objection was that this is a cross-sectional study without a control group and without a baseline assessment. This allows us to conclude that there is an association between the intervention and improved outcomes. Another potential weaknesses in the study could be other effects that may have contributed to the improved outcomes over 12 years like changing economic conditions, better conditions in antenatal care and general medical care, greater access to care not ruled by the referral system. Instead of these limitations it is our aim to encourage other groups to develop similar programs.

Our results are in accordance to previous study that reorganization of health system was of great value to eliminate inequality in health assistance improving health outcomes and results in lower PMR [18]. The political and administrative system and the organizational structure will strongly affect operations and, in turn, service outputs. Subsequently, this will have a direct effect on the health of women and newborns [19,20]. The organizational structure of safe pregnancy services [19,21] (including service infrastructure, sectorial integration, service delivery strategies and partners) and safe pregnancy practices (including management supervision, training, commodities acquisition/distribution, research and evaluation, and transport) are all included in our program. Thus, our referral program could be considered for use as an intervention for improving the safety of pregnancy.

Governments have a long history of announcing lofty and well-meaning pledges to make the world a healthier place. The dominant model for improving public health focuses almost exclusively on the supply side of the health equation by improving the quality of services, expanding coverage, and telling people why they should use the health service and where it is available [2]. With our program, a two-pronged approach was used: on the supply side, the health infrastructure was upgraded, while demand was increased [22]. There is an increasing consensus that stronger health systems are keys to improving health outcomes [2].

This program could be considered a Safe Motherhood Model (SMM) [23], i.e., a program to assist in effectively allocating the resources associated with reducing the maternal mortality ratio. The adequacy of delivery care, the access to services and the social and economic content have a direct influence on safe motherhood [14]. National programs to improve maternal and neonatal health are wide ranging, and they involve very large investments. However, uniform, periodic measurements of the levels and types of effort being made are rare. This report uses a methodology that covers a twelve-year period by component and region, with attention to specific criteria [20].

Regionalization provided a framework for in uterus or postnatal transfer of high-risk mother-perinatal dyads to the level of care that offered them the best chance for survival [24].

In conclusion, our results demonstrate the importance of prioritizing the reorganization of referral systems and are in agreement with the conclusions of Ronsmans et al. [25] reductions in perinatal mortality will require strategies such as early detection and management of health problems during pregnancy. This strategy supports the hypothesis that a safe motherhood referral system for antenatal/intrapartum assistance is a tool to ensure mothers and their infants survive during these crucial periods.

## Conclusions

These results demonstrate the importance of prioritizing the reorganization of referral systems and are in agreement with the conclusions of Ronsmans et al. [25] reductions in perinatal mortality will require strategies suchas early detection and management of health problems during pregnancy. This strategy supports the hypothesis that a safe motherhood referral system for antenatal/intrapartum careis associated with an increase in vaginal deliveries and a decrease in C-sections and PMR. In addition, in the present study, this system was responsible for the shift from direct to indirect causes of maternal death.

## Details of ethics approval

This study was approved by the Ethics Committee of Botucatu Medical School, Sao Paulo State University-UNESP (project number 2048/2006).

## Competing interests

The authors declare that they have no competing interests.

## Authors' contributions

MVCR and IMPC had the original idea for the study. MVCR, IMPC, IM, PMSSM, CVCR and GM were responsible for data collection and the plan of analysis. All authors saw the output of analysis drew the tables and commented on their significance. IM wrote the first version of the manuscript, then reviewed, amended and corrected by MVCR, IMPC and SSW. All authors read the final version of the manuscript and agreed with its content before submission.
